# Changes of *Fusarium oxysporum f.sp. lactucae* levels and soil microbial community during soil biosolarization using chitin as soil amendment

**DOI:** 10.1371/journal.pone.0232662

**Published:** 2020-05-05

**Authors:** Tara E. Randall, Jesus D. Fernandez-Bayo, Duff R. Harrold, Yigal Achmon, Kelley V. Hestmark, Thomas R. Gordon, James J. Stapleton, Christopher W. Simmons, Jean S. VanderGheynst

**Affiliations:** 1 Department of Biological and Agricultural Engineering, University of California, Davis, CA, United States of America; 2 Department of Food Science and Technology, University of California, Davis, CA, United States of America; 3 Department of Plant Pathology, University of California, Davis, CA, United States of America; 4 Statewide Integrated Pest Management Program, University of California, Kearney Agricultural Research and Extension Center, Parlier, CA, United States of America; 5 Department of Bioengineering, University of Massachusetts, Dartmouth, MA, United States of America; Tallinn University of Technology, ESTONIA

## Abstract

Regulatory pressure along with environmental and human health concerns drive the development of soil fumigation alternatives such as soil biosolarization (SBS). SBS involves tarping soil that is at field capacity with a transparent film following amendment with certain organic materials. Heating via the greenhouse effect results in an increase of the soil temperature. The organic amendments can promote microbial activity that can enhance pest inactivation by depleting oxygen, producing biopesticidal fermentation products, and competing with pests. The properties of the organic amendments can heavily influence the type and magnitude of these effects. This study evaluated the viability of chitin as a novel SBS soil amendment to influence soil fungal and bacterial microbial communities, including control of the plant pathogen *Fusarium oxysporum* f.sp. *lactucae* (FOL). Changes to FOL and the broader soil microbiota were monitored in response to biosolarization using 0.1% (by dry weight) amendment with chitin (Rootguard). FOL suppression was only observed in chitin amended soils that were incubated at room temperature, not under solarized conditions. Conversely, it decreased solarization efficacy in the upper (0–10 cm) soil layer. The presence of chitin also showed increase in FOL under anaerobic and fluctuating temperature regime conditions. Biosolarization with chitin amendment did exhibit an impact on the overall soil microbial community. The fungal genus *Mortierella* and the bacterial family *Chitinophagaceae* were consistently enriched in biosolarized soils with chitin amendment. This study showed low potential FOL suppression due chitin amendment at the studied levels. However, chitin amendment showed a higher impact on the fungal community than the bacterial community. The impact of these microbial changes on crop protection and yields need to be studied in the long-term.

## Introduction

Soil-borne pathogens and pests are a major and complex issue inhibiting agricultural production [1, 2]. Pre-plant chemical fumigation is the most effective way to control soil-borne pathogens, however, owing to cost and regulatory constraints, this practice is no longer an option for production of most crops [3, 4]. For this reason, it is critical that effective and environmentally sustainable alternatives to chemical fumigation are developed.

In 2015, lettuce generated nearly 1.9 billion dollars in revenue, making it one of the most valuable vegetable crops in the United States [5]. Lettuce can be severely damaged by Fusarium wilt, a disease caused by the pathogenic fungus, *Fusarium oxysporum* f. sp. *lactucae* (FOL) [6]. Since its discovery in Japan in 1955 [7], FOL has been observed across Northern America[6], South America [8], Europe [9], and East Asia [10]. One study found that a 0.5% *Fusarium* disease severity corresponded to a revenue loss of $11.66 per acre, while a 2.2% *Fusarium* disease severity corresponded to a revenue loss of $100.85 per acre [11]. Accordingly, management of Fusarium wilt is a priority for lettuce growers where this disease occurs.

Soil biosolarization (SBS) is a soil disinfestation process in which soil is amended with organic matter prior to solarization. During solarization, soil is tarped with transparent film during summer months which results in elevated soil temperature due to the accumulation of energy in soil as a result of the greenhouse effect [12]. The microbial activity that occurs during metabolism of exogenous organic carbon results in anaerobic conditions [13], accumulation of organic acids [14, 15] and changes in the microbial community [16, 17] that may potentially enhance pathogen inactivation. The characteristics of the organic amendment play a key role in the efficacy and mechanism of pathogen control. Given the vast diversity of biomass resources that could be used as amendment for SBS, is it is important to understand how biomass composition and concentration in soil impacts SBS efficacy.

In this study, chitin was tested as an amendment for SBS. Chitin is a structural element found in the exoskeletons of arthropods, like crustaceans, and in fungal cell walls. [18]. Global shellfishery waste generation ranges from 6 to 8 million tons annually, with crustacean waste often being disposed of in landfills or the ocean [19]. Harvesting shellfish is not only wasteful, but expensive. In Australia, it can cost up to $150 USD to dispose of one ton of shellfish waste [20]. Chitin comprises 20–50% of the dry-weight of shellfish waste [21]. If the crustacean-produced chitin could be harnessed for SBS, it would not only reduce waste but could also be a viable replacement to synthetic chemical soil fumigants. The microbial degradation of chitin may result in the release of ammonia (NH_3_), which is toxic to nematodes and fungi [22]. In addition, because chitin is an essential part of fungal cell walls [23], the availability of chitin during SBS may promote activity of chitinolytic microorganisms that are capable of hydrolyzing fungal cell walls. Consistent with this expectation, it has also been observed that chitinolytic microorganisms can inhibit the growth of many fungal pathogens that threaten global crop production [24].

Aside from the potential inhibition of pathogenic fungal disease, many studies have reported beneficial effects of chitin amendment on plant growth. Debode et al showed that chitin amendment increased the fresh yield weight of lettuce leaves as well as the fungal and bacterial biomass in the lettuce rhizosphere [25]. Another study found similar findings, that chitin treated plants had increased in vitro fresh weight as well as radicle length and total carbon and nitrogen content [26]. Cretoiu et al reported that chitin addition beneficially changed the soil microbiota in the soil rhizosphere effecting plant physiology and microorganisms’ ability to colonize the plant root structures [27]. Chitin amendment has also been shown to trigger plant immunity as the biopolymer acts as a pathogen-associated-molecular-pattern (PAMP) [25, 28]. Although the beneficial effects of chitin amendment have been demonstrated, amending soil with only chitin prior to SBS has not been studied. The presence of chitin may enhance chitinolytic microbial populations that, if able to survive the extreme conditions during SBS, might significantly enhance the disinfestation of fungal soil-borne pathogens.

The objective of this study was to assess the utility of chitin as an SBS soil amendment to control *Fusarium oxysporum* f. sp. *lactucae* (FOL), the causal agent of Fusarium wilt of lettuce, and to measure changes induced in the overall soil microbial community. Field studies were performed to measure the efficacy of chitin amendment on SBS for the control FOL, while laboratory studies were performed to simulate and understand how temperature and oxygen conditions occurring during treatment may inhibit or enhance fungal inactivation and affect soil microbiota.

## Materials and methods

### Field trial

The field experiment was conducted at the UC Davis Plant Pathology Research Farm where infestation with FOL was achieved by incorporating sand inoculum into soil to a depth of 25 cm. Thereafter, the entire field was planted with a mix of lettuce cultivars that were susceptible to Fusarium wilt. On reaching maturity, lettuce was incorporated into the soil. This cropping cycle was repeated two additional times, resulting in at a high level of inoculum throughout the field [29]. The field soil was sandy clay loam (47%, 27% and 26% of sand, silt and clay, respectively), the organic matter (OM) content was 2.64% and the field capacity was 21.90% (wet basis). Prior to initiation of the treatments, the field was rototilled and flattened. The experimental site was prepared as described elsewhere [15]. Large (60x22 cm) PVC pipes, for non-amended soil and small (60x7.6 cm) PVC pipes for chitin-amended soil were embedded in each plot. They served to isolate the mesocosms, allowing gas and water exchange between the mesocosms and the natural soil from below. The top 20 cm of soil in the columns was removed to accommodate the mesocosms. Mesocosms (20 cm tall and 5 cm and 15 cm in diameter, for small and large systems, respectively) were prepared as experimental units. To prepare the mesocosms FOL-infested field soil was sieved at 3 mm. The large mesocosms were filled with non-amended field soil and the small mesocosms were filled with the soil amended with chitin (Rootguard) at 0.1 wt% (dry basis) amendment rate, following manufacture instructions. The same amended and non-amended treatments were incubated in parallel at room temperature (RT, 23±1°C). Five replicates were prepared for each treatment. Temperature loggers (Thermochron iButtons model 1922L, Embedded Data Systems, Lawrenceburg, KY) were embedded at 7 and 15 cm depths in non-amended soil treatments to log temperature every ten minutes during the trial. To measure initial levels of FOL, five mock-replicates were also prepared for each treatment and placed into 250 mL high-density-polyethylene (HPDE) containers. Prior to the experiment, samples were equilibrated to field capacity moisture content in refrigerated conditions (4°C) overnight.

The day of the field trial, mesocosms with time zero samples were directly stored for later analysis. The insulated-mesocosms were randomly placed into the previously embedded field PVC pipes in the field plot. Plots were then drip irrigated for one hour before being covered with 0.7 mil transparent plastic (Husky Film Sheeting; Poly-America, Inc., Grand Prairie, TX). After the 8-day solarization trial was completed, the tarp was removed and soil in each mesocosm was separated into two samples by depth (0–10 cm and 10–20 cm). From each of the two depths, soil was homogenized, separated and stored for further analyses. Samples used for DNA extraction were placed in a -80°C freezer, while samples used for FOL inactivation analyses were left at room temperature to air dry. Once desiccated, the samples were refrigerated (4°C).

### Laboratory incubations

Laboratory studies were performed to examine the role of chitin amendment, oxygen, and temperature on FOL inactivation in a controlled soil environment. Soil collected from the field was amended at the same rate as in the field experiment (0.1 wt%, dry basis), and wetted to the moisture content measured in the mesocosms at the end of the field trials (21 wt%, wet basis). For each test, samples were prepared in triplicate with 150g of soil per sample.

To test the effect of oxygen conditions, one set of samples (non-amended and chitin-amended) was incubated under anaerobic conditions by sealing in plastic bags (Ziploc Quart Freezer Bags; SC Johnson, Racine, Wisconsin) and another set was incubated under aerobic conditions using open 250mL HPDE bioreactors. To test the impact of temperature, samples were incubated at a constant temperature of 30°C and under a fluctuating temperature regime (30°C for 12h, 40°C for 12h) to simulate field temperature conditions. These temperatures were chosen as they are likely to be found at lower depth soil layer during SBS and were enough to weaken FOL but not to kill it [6], allowing to observe any potential chitin effect. The incubations lasted 8 days.

### Analysis of FOL in the soil

FOL levels in soil samples from the field and laboratory experiment were quantified using dilution plating as previously described [6]. Soil samples were prepared by sieving them to 4 mm or smaller after drying then 20 grams per sample were suspended in 200 mL of 1% sodium hexametaphosphate. After 5 minutes of stirring, 10 mL of solution was transferred into 90 mL of 0.1% water agar. After an additional 5 minutes of stirring, 400 μl were spread over the surface of each of 12 plates containing Komada’s selective medium, prepared as previously described [30]. Inoculated plates were left to incubate at room temperature (23±2°C) under fluorescent light continuously for 10 to 11 days. After incubation, FOL colonies were identified based on the colonies’ distinct morphology as described by Scott et al. [6] Results were expressed as colony forming units (CFU) per gram of soil.

### DNA extraction and sequencing from soil samples

DNA was extracted from soil from laboratory experiments using a Powersoil DNA Isolation Kit (MO-BIO, Carlsbad, CA). Each sample had duplicate extractions and the DNA was pooled at the final step of the extraction. Sequences corresponding to the internal transcriber spacer (ITS) and 16S rRNA gene were amplified from DNA extracts using polymerase chain reaction (PCR). Sequencing of the V4 hypervariable region of the broadly conserved 16S rRNA and internal transcribed spacer (ITS) region for bacterial and fungal taxonomic identification, respectively, was performed on purified DNA by the United States Department of Energy Joint Genome Institute using the Illumina MiSeq platform, as previously described [31]. The raw Illumina sequencing data were deposited at the National Center for Biotechnology Information (NCBI) under the BioProject accession PRJNA599426.

### Data analysis

Microsoft® Excel (Microsoft Inc., Bellevue, WA) and JMP-IN software (version Pro 12, SAS, Cary, NC) were used for all statistical analyses. One-way and multiway ANOVA, Tukey’s Honestly Significant Difference test (Tukey’s HSD) and Student’s t-Test analyses were used to determine statistically significant effects and differences among amendment, temperature, and oxygen conditions in field trials and in laboratory incubations. Statistical significance was set at the 95% confidence level. Data analysis was done after removing singletons from the OTU data. R-Studio (RStudio Version 1.1.423 –© 2009–2018 RStudio, Inc.) was used to estimate and show relative abundance (RA) of OTUs. Non-metric multidimensional scaling (NMDS) plots of the communities in each sample were done using the vegan package in R-Studio [32]. SIMPER function was used to measure similarity percentages between samples.

## Results

### FOL results under field conditions

**Fig 1:**
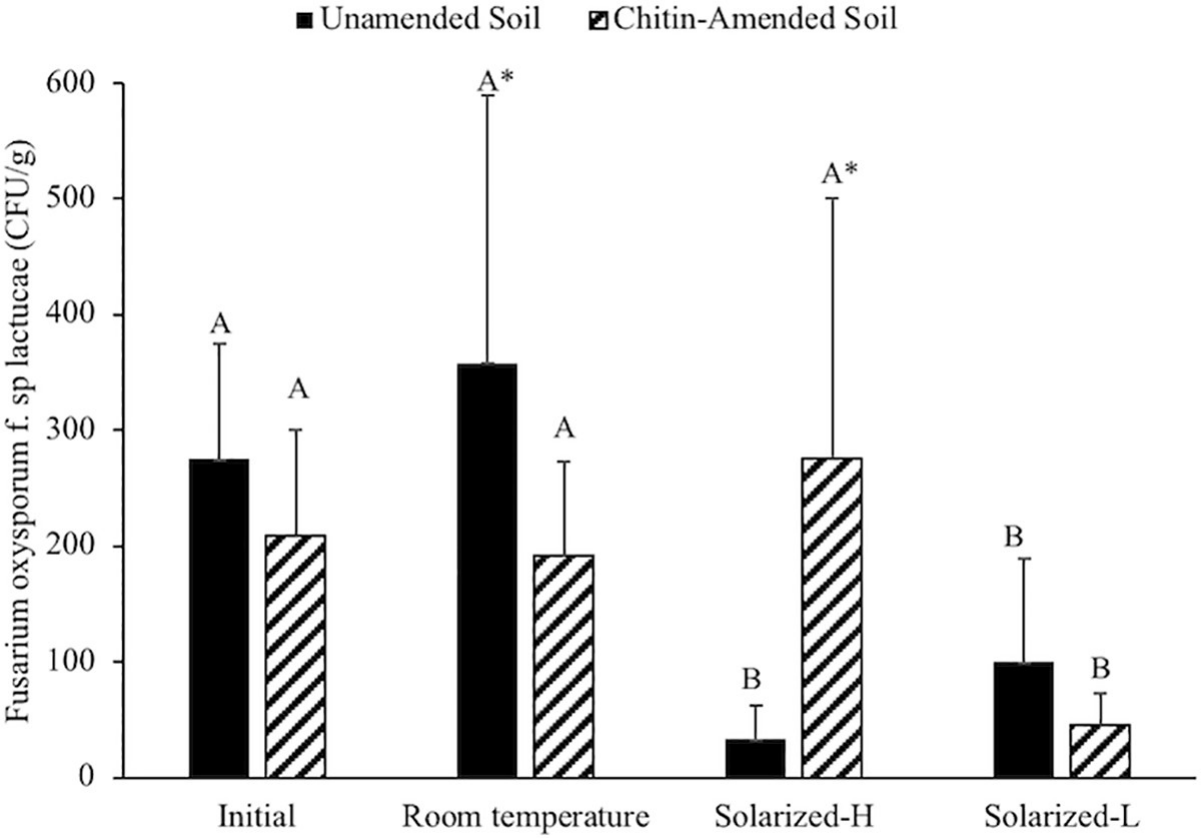
Average *Fusarium oxysporum* f. sp *lactucae* (CFU/g of soil) under solarized field conditions. Capital letters denote significant differences in the log transformed CFU in the HSD Tukey test within the same amendment for the different incubation regimes, whereas stars denote significant differences in the t-test within the same incubation regime between amendment type (P<0.05).

During biosolarization, mean maximum temperature of the non-amended soil was 46.52±0.74°C and 38.18±0.49°C, and mean minimum temperature was 23.50±0.82°C and 27.86±0.43°C at the top (0–7 cm) and bottom (14–20 cm) layers, respectively. The multiway ANOVA testing the effect of incubation regime (non-solarized, incubated at RT and solarized bottom and top soil layers), amendment type (non-amended and chitin amended) and their interaction in the log transformed FOL showed a significant effect of the incubation type and the interaction between incubation type and amendment (P<0.05, S1 Table). In the non-amended samples, the Tukey’s HSD test showed that the solarized samples at both the lower (10–20 cm) and upper (0–10 cm) layers had significantly lower CFUs than the initial and RT-incubated samples (P<0.05, Fig 1). The chitin-amended samples in the lower, solarized layer showed significant the lowest CFU (P<0.05). Grouping by incubation regime, FOL CFU levels were significantly lower (P<0.05) in the chitin-amended than in the non-amended soil at RT, whereas, the top-solarized layer showed an opposite trend; FOL CFUs in the chitin-amended samples were significantly higher than in the non-amended samples (P<0.05).

### FOL results under controlled environmental laboratory conditions

**Fig 2:**
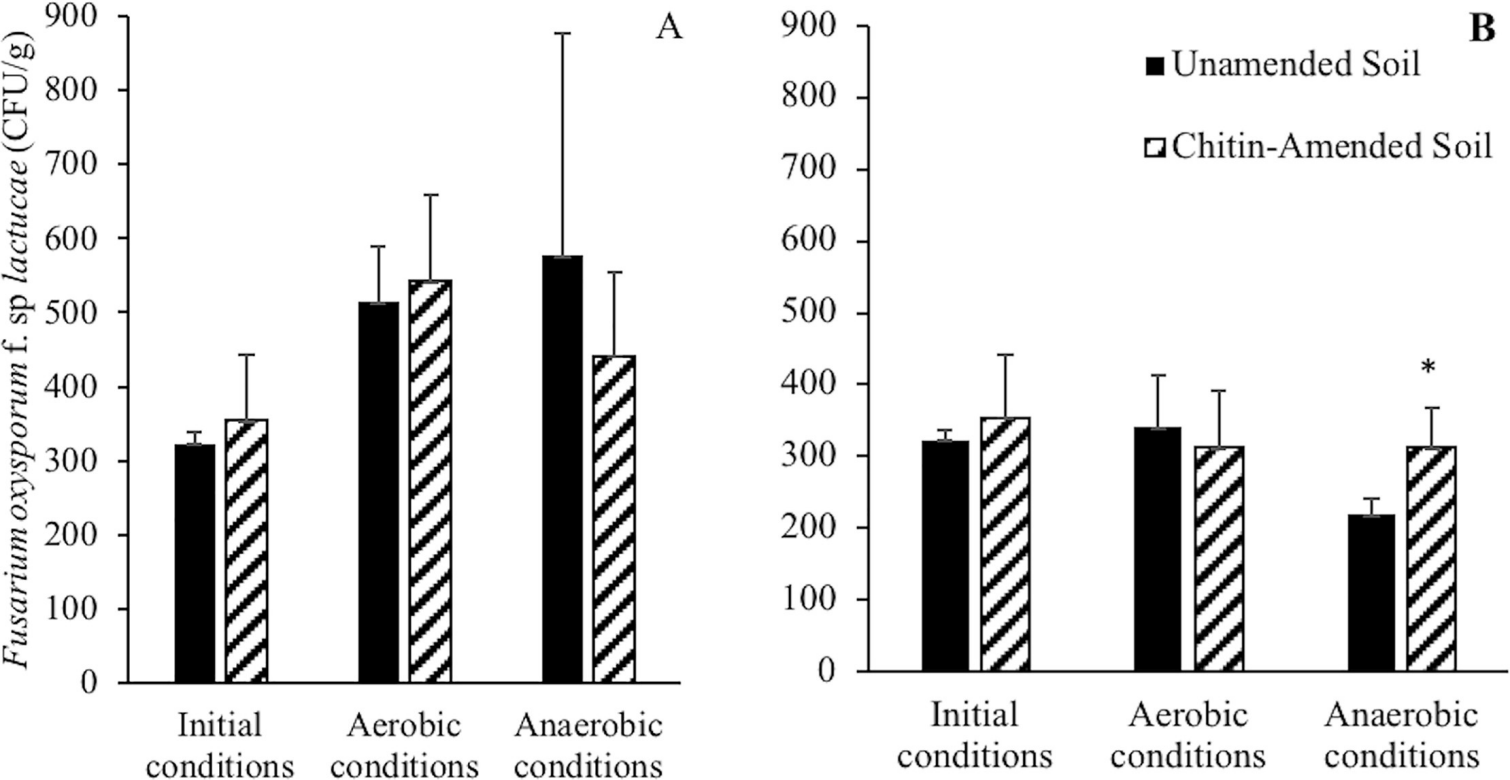
Average *Fusarium oxysporum* f. sp *lactucae* (CFU/g) of the soil samples incubated in the laboratory at constant temperature (A) and fluctuating conditions (B). The star denotes significant differences in the log transformed CFU (P<0.05) between non-amended and chitin-amended soil.

The effect of the interaction between chitin amendment and soil environmental parameters on FOL concentration in soil was further studied in laboratory experiments, under controlled temperature and oxygen conditions. Multifactorial ANOVA of amendment type (chitin-amended, non-amended), incubation regime (initial, aerobic, anaerobic) and temperature regime (constant at 30°C and fluctuating between 30°C and 40°C) and their interaction on the log transformed FOL CFUs showed significant effects only for temperature regime and the interaction between incubation and temperature regime (P<0.05, S2 Table). Aerobic and anaerobic incubation showed significantly higher FOL CFUs under the constant temperature regime than the fluctuating regime (P<0.05, Fig 2). The CFU levels in chitin-amended soil incubated under anaerobic conditions and the fluctuating temperature regime were significantly higher (P<0.05, Fig 2B) than the non-amended soils.

### Impact of chitin amendment on microbial diversity during controlled environmental laboratory conditions

#### Fungal community diversity

**Fig 3:**
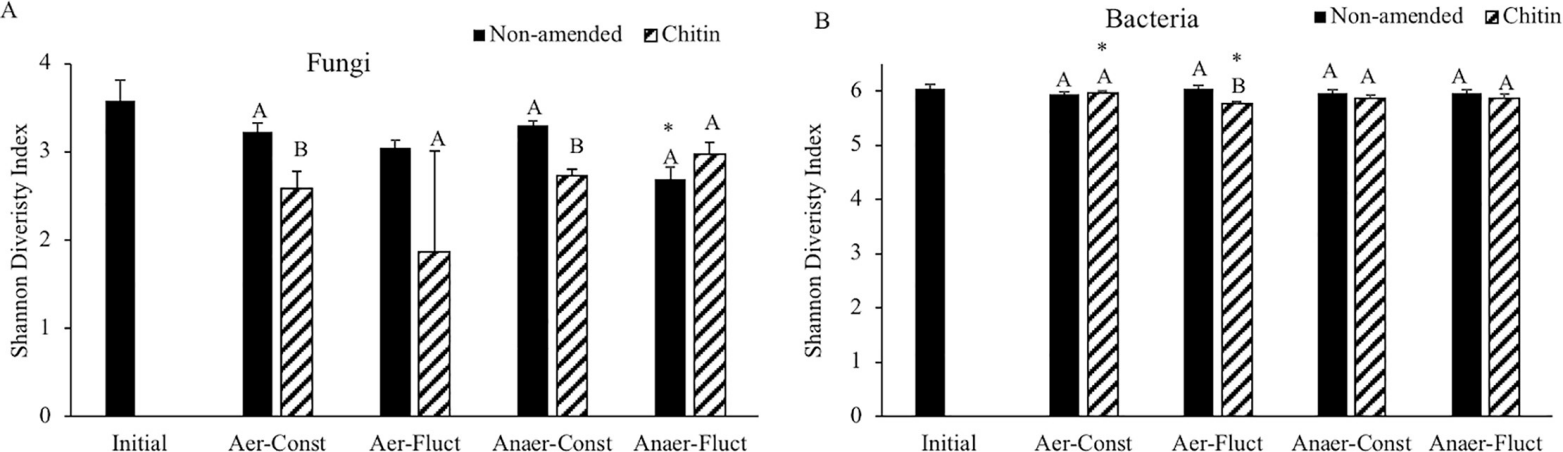
Average Shannon diversity index of the Fungi (A) and Bacteria (B) communities in the soil samples incubated in the laboratory at constant or fluctuating temperature and at aerobic or anaerobic conditions. Different letters denote significant differences (P<0.05) between non-amended and chitin-amended soil at each incubation regime. Stars denote significant differences between incubation regimes within the same amendment (P<0.05).

**Fig 4:**
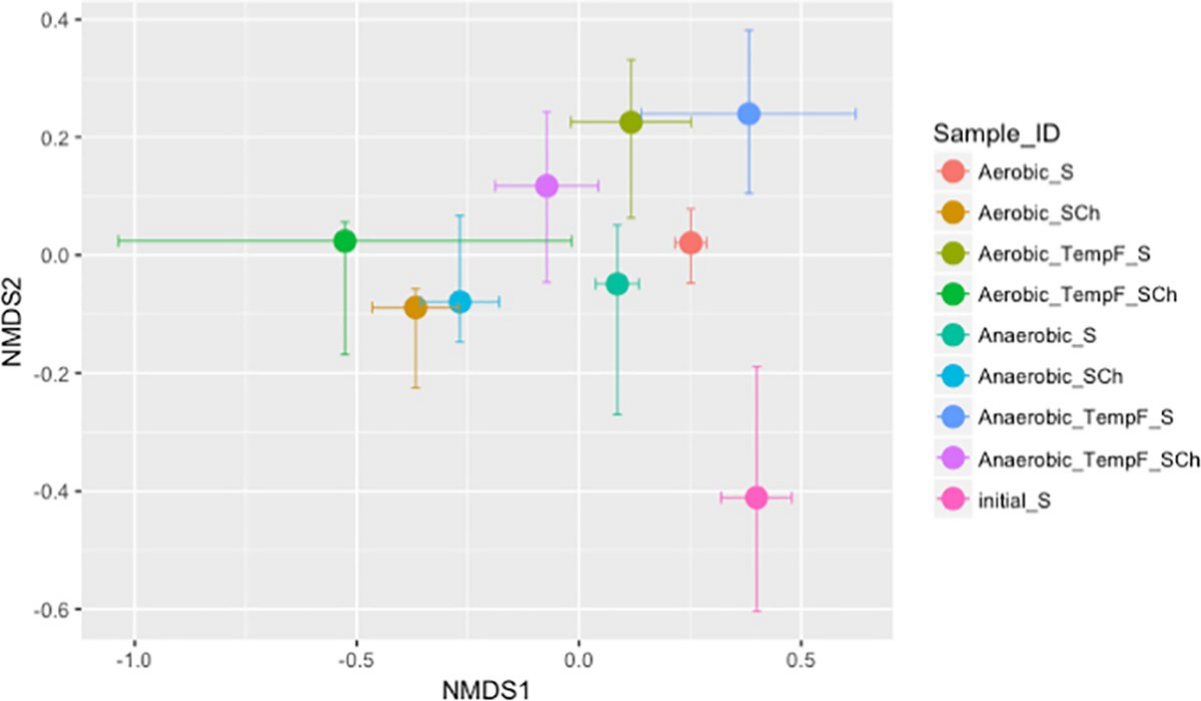
NMDS plot of the fungal community incubated in the lab under different aeration and temperature regimes. S = Non-Amended; SCh = Chitin amended; TempF = Fluctuating temperature.

Only the Shannon diversity index for fungal microbial diversity (Fig 3) was significantly affected by soil amendment type (S3 Table, P<0.05). Furthermore, only the chitin-amended samples had significantly lower Shannon diversity index than non-amended samples when incubated at constant temperature and under both the aerobic and anaerobic oxygen regimes (P<0.05, Fig 3A). Within the same amendment type, non-amended samples incubated under anaerobic conditions and fluctuating temperature showed significantly lower Shannon diversity index than amended samples (P<0.05). The non-metric multidimensional scaling (NMDS) plot (Fig 4) showed shifts in the fungal microbial community due to the incubations. These shifts showed grouping of samples by amendment type, but the differences between microbial communities were not marked. Grouping was more affected by temperature regimes (Fig 4) than by aeration regimes (Fig 4).

#### Bacterial community diversity

**Fig 5:**
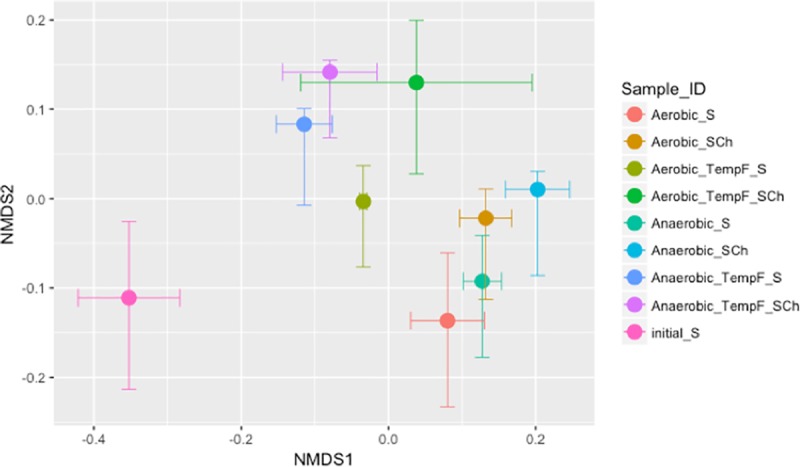
NMDS plot of the bacterial community incubated in the lab under different aeration and temperature regimes. S = Non-Amended; SCh = Chitin amended; TempF = Fluctuating temperature.

The bacterial community showed higher Shannon diversity index values than the fungal community (Fig 3B). Multiway ANOVA showed significant effects of amendment addition and the interaction between temperature regime and amendment (S3 Tabe, P<0.05). The comparison between chitin-amended and non-amended samples for each incubation regime showed significantly lower bacterial diversity in the chitin-amended samples when incubated under aerobic and fluctuating temperature conditions (P<0.05). Within the chitin-amended samples, those incubated under aerobic conditions showed a significantly higher Shannon index when the temperature regime was constant compared to fluctuating temperature conditions (P<0.05). The NMDS plot (Fig 5) also showed shifts in the bacterial community due to incubation compared to the original soil. The effect of the temperature regime was more evident for bacteria than for fungi.

### Effect of incubation regime and chitin on soil fungal phyla

The most abundant phyla (mean relative abundance >5%) revealed by the sequencing of the ITS region were Basidiomycota, Chytridiomycota, Zygomycota, Ciliophora (Protozoans), Chlorophyta (Algae) and Ascomycota (Table 1). The most abundant fungal phylum in the orignal soil was Basidiomycota (35.7%, Table 1). Multiway ANOVA analysis showed significant effects of amendment, temperature and aeration regime on abundance of this phylum (P<0.05). The relative abundance of the Basidiomycota was negatively affected by the presence of chitin, aerobic conditions and constant temperature. Among the incubated samples this phylum was significantly enriched in the non-amended samples incubated under anaerobic and fluctuating temperature conditions when compared with the original soil and with the chitin-amended samples incubated under any condition, except those incubated under anaerobic and temperature fluctuating conditions (P<0.05). Chytridiomycota was the second most abundant phylum in the original soil (18.8%) and no significant effect of the factors was observed, however, any incubation regime significantly (P<0.05) decreased its relative abundance in all samples compared to the initial sample except for non-amended samples incubated at constant temperature and aerobic conditions and chitin-amended samples incubated under anaerobic and fluctuating temperature condition.

**Table 1:** Relative abundance and standard deviation (%) of the six most abundant fungal phyla encountered under controlled laboratory conditions. Different letters denote significant differences between treatment (same row) for each phylum (P<0.05).

Temperature regime	Amendment	Incubation	Basidiomycota	Chytridiomycota	Zygomycota	Ciliophora	Chlorophyta	Ascomycota
None	None	Initial	35.7±0.96b	18.79±12.44a	13.24±3.41c	12.98±2.17abc	11.69±6.82a	4.93±1.31a
Constant	None	Aerobic	39.29±5.6ab	6.55±4.04ab	17.16±1.75bc	21.31±2.74a	7.54±0.39a	6.82±3.5a
Constant	Chitin	Aerobic	26.4±6.26b	3.92±1.47b	47.69±7.34ab	11.37±2.51bc	5.41±0.5a	3.99±1.09a
Fluctuating	None	Aerobic	45.33±4.4ab	4.43±0.52b	14.78±6.43c	13.05±1.15abc	10.31±3.23a	9.67±4.87a
Fluctuating	Chitin	Aerobic	22.61±17.58b	3.17±2.61b	58.2±30.7a	5.48±3.52c	5.91±4.11a	3.81±3.23a
Constant	None	Anaerobic	38.21±5.53ab	3.61±1.5b	21.13±2.52bc	19.02±7.27ab	8.41±0.49a	7.52±3.61a
Constant	Chitin	Anaerobic	31.48±5.03b	3.86±1.92b	37.22±1.8abc	15.25±1.92ab	6.46±2.36a	4.82±1.81a
Fluctuating	None	Anaerobic	60.24±10.42a	3.14±1.62b	11.33±4.71c	9.62±3.33bc	10.97±0.64a	3.23±0.52a
Fluctuating	Chitin	Anaerobic	39.32±6.26ab	6.41±4.37ab	26.43±7.93abc	9.89±0.09bc	11.05±4.64a	4.4±1.26a

Zygomycota had a relative abundance in the original soil of 13.4%. The multiway analysis showed a significant effect of amendment, aeration regime and their interaction (P<0.05) and the phylum was enriched by the presence of chitin and aerobic conditions. The original soil and the non-amended samples incubated in fluctuating temperature had the lowest relative abundance (P<0.05). The relative abundance of Zygomycota was significantly higher (P<0.05) in the chitin-amended samples incubated under aerobic conditions. Ciliophora had relative abundance similar to Zygomycota in the origianl soil (13.0%). Relative abundance of Ciliophora was significantly affected by amendment type, temperature and the interaction between amendment type and aeration regime (P<0.05). The relative abundance of Ciliophora significantly decreased in all the chitin-amended soils except those incubated under anaerobic and constant temperature conditions (P<0.05). The phylum Chlorophyta had relative abundance similar to Zygomycota and Ciliophora in the original soil (11.7%). The temperature regime had a significant effect (P<0.05) on this phylum, however, Tukey’s HSD test did not show significant differences between samples (Table 1). Ascomycota had a relative abundance of 4.9% in the original soil and there was only a slight negative effect on the relative abundance when samples were amended with chitin (P<0.05). Also, Tukey’s HSD test did not show significant differences between samples (Table 1).

### Influence of incubation regime and chitin on soil bacteria phyla

The most abundantly revealed phyla in the bacterial community (mean relative abundance >5%) were Proteobacteria, Bacteroidetes, Acidobacteria, Chloroflexi, Actinobacteria and Gemmatimonadetes. Proteobacteria was the most abundant bacteria phylum in the original soil (29.0%, Table 2). Proteobacteria relative abundance was significantly affected by the amendment type and aeration rate (P<0.05, S4 Table). After the experimental incubation period, only the non-amended samples incubated at constant temperature under aerobic conditions showed significantly lower relative abundance of Proteobacteria than the chitin-amended samples incubated at constant temperature and anaerobic conditions. Bacteroidetes showed a relative abundance of 16.7% in the original soil. Multiway ANOVA showed a significant effect of the aeration and temperature regimes and their interaction (P<0.05). Samples incubated under fluctuating temperature and anaerobic conditions showed lower relative abundance. Acidobacteria had a relative abundance of 9.3% in the original soil. The amendment and temperature regimes were the main factors affecting Acidobacteria relative abundance. Both non-amended samples incubated at constant temperature showed significantly higher relative abundance than the original soil (P<0.05). Phylum Chlorofexi was significanlty enriched from 6.7% in the original soil to >10.3% in the non-amended samples incubated at fluctuating temperature (P<0.05). As with phylum Acidobacteria, amendment and temperature regimes were the main factors affecting the relative abundance of Choroflexi. The relative abundance of Actinobacteria in the original soil also was similar to Acidobacteria levels (9.4%). In this case, incubation significanly decreased Actinobacteria relative abundance when samples were incubated at constant temperature (P<0.05). Multiway ANOVA confirmed temperature regime and its interaction with the incubation regime to be significant effects (P<0.05). Finally, Gemmatimonadetes had an initial a relative abundance of 3.3%. Multiway ANOVA revealed that this phylum was significantly affected by the three studied factors (P<0.05), showing a significant increase (P<0.05) in relative abunance in all incubated samples.

**Table 2:** Relative abundance and standard deviation (%) of the six most abundant bacterial phyla encountered under controlled laboratory conditions. Different letters denote significant differences between treatment (same row) for each phylum (P<0.05).

Temperature regime	Amendment	Incubation	Proteobacteria	Bacteroidetes	Acidobacteria	Chloroflexi	Actinobacteria	Gemmatimonadetes
None	None	Initia	28.97±2.29abc	16.69±1.98abc	9.35±1.42b	6.79±0.38c	9.44±1.28a	3.26±0.35c
Constant	None	Aerobic	26.21±2.05c	18.34±1.22ab	12.96±2.36a	8.03±0.4bc	5.76±1.24c	6.95±0.31a
Constant	Chitin	Aerobic	29.36±1.73abc	19±2.52a	10.51±0.68ab	6.98±0.28c	6.84±0.81bc	6.59±0.08ab
Fluctuating	None	Aerobic	26.53±0.7bc	16.3±2.58abc	9.89±0.09ab	10.51±0.71a	7.2±0.77abc	7.12±1a
Fluctuating	Chitin	Aerobic	27.58±1.05abc	18.5±4.3ab	8.32±0.71b	9.18±0.2ab	8.05±0.57abc	5.43±0.31b
Constant	None	Anaerobic	27.39±1.09abc	18.52±0.78ab	12.97±1.05a	7.44±0.28c	5.83±0.84c	6.58±0.17ab
Constant	Chitin	Anaerobic	31.18±0.98a	18.92±1.67a	10.52±0.16ab	7.52±0.28bc	5.82±0.3c	6.03±0.06ab
Fluctuating	None	Anaerobic	28.95±1.65abc	11.99±0.91c	8.8±0.86b	10.27±0.81a	9.06±0.62ab	5.88±0.46ab
Fluctuating	Chitin	Anaerobic	30.49±0.16ab	12.39±1.18bc	8.08±0.34b	9.74±1.2a	9.04±0.88ab	5.48±0.55b

### Similarity percentage (SIMPER) analysis of the ten OTUs that contributed the most to differences between the microbial communities of non-amended and chitin-amended samples

#### Fungal community

The total contribution of the ten most important OTUs contributing to the different fungal communities in the comparison between the chitin-amended and non-amended soil under all the incubation regimes tested in the laboratory was >59%. The genus OTU *Mortierella* was the organism that contributed the most to the difference between non-amended and chitin-amended samples in all incubation regimes (Table 3), and was consistently enriched via chitin amendment. The OTU *Cryptococcus* was highly enriched in the non-amended samples incubated under aerobic conditions in both temperature regimes. However, under anaerobic conditions, higher abundances of *Cryptococcus* were observed in chitin-amended samples under constant temperature incubations compared to fluctuating temperatures. The OTU of the family *Psathyrellaceae* was higher in the non-amended samples under all incubation regimes. A similar trend was observed for both OTUs of the phylum Ciliophora, except in the samples incubated under anaerobic and fluctuating temperature conditions. The OTU *Minimedusa* was also higher in the chitin-amended samples incubated in under aerobic conditions compared to anaerobic conditions. The OTU Ascomycota showed higher relative abundances in the non-amended samples than in the chitin-amended samples incubated under aerobic and fluctuating temperature conditions and in anaerobic and constant temperature conditions. Finally, other remarkable OTUs that showed higher relative abundance in the presence of chitin were *Bolbitiaceae* and *Rhizophlyctis* in samples incubated under anaerobic conditions and fluctuating temperature. On the other hand, other remarkable OTU enriched in the non-amended samples included that of the order Agaricales under anaerobic conditions and fluctuating temperature.

**Table 3:** Cumulative contribution (Cum,%) of the SIMPER analysis of the main OTUs that contributed the most to the differences in the microbial community between non-amended (Non_Chi, %) and chitin (Chitin, %) amended samples under different incubation regimes.

Aerobic and constant temperature				Aerobic and fluctuating temperature				Anaerobic and constant temperature				Anaerobic and fluctuating temperature			
OTU	Chitin	Non_Ch	Cum (%)	OTU	Chitin	Non_Ch	Cum (%)	OTU	Chitin	Non_Ch	Cum (%)	OTU	Chitin	Non_Ch	Cum (%)
*g*:*Mortierella*	42.28	6.93	34.10	*g*:*Mortierella*	55.38	10.38	40.75	*g*:*Mortierella*	31.56	10.89	23.15	*g*:*Mortierella*	21.11	6.01	16.62
*g*:*Cryptococcus*	17.10	26.72	43.38	*g*:*Cryptococcus*	18.26	31.58	53.15	*g*:*Cryptococcus*	5.51	0.08	29.25	*o*:*Agaricales*	0.05	12.45	30.29
*g*:*Minimedusa*	6.31	1.79	48.15	*f*:*Psathyrellaceae*	0.14	9.02	61.20	*f*:*Psathyrellaceae*	0.05	4.96	34.75	*f*:*Psathyrellaceae*	0.02	11.60	43.06
*g*:*Mortierella*	2.90	6.98	52.08	*p*:*Ascomycota*	0.56	3.76	64.10	*p*:*Ascomycota*	3.60	7.16	40.20	*g*:*Cryptococcus*	28.41	30.68	52.10
*p*:*Ciliophora*	4.24	6.93	55.81	*p*:*Ciliophora*	2.50	4.52	66.54	*p*:*Ciliophora*	21.54	25.28	45.45	*f*:*Bolbitiaceae*	4.94	0.11	57.51
*f*:*Psathyrellaceae*	0.16	3.88	59.44	*p*:*Ciliophora*	0.35	2.00	68.20	*p*:*Ciliophora*	2.11	5.16	48.87	*g*:*Rhizophlyctis*	2.87	0.00	60.68
*g*:*Laetisaria*	0.01	2.85	62.18	*p*:*Ascomycota*	0.21	1.76	69.85	*p*:*Ascomycota*	2.16	3.65	52.11	*g*:*Minimedusa*	2.10	0.66	63.08
*p*:*Ciliophora*	0.38	2.84	64.64	*f*:*Psathyrellaceae*	1.71	0.01	71.41	*f*:*Psathyrellaceae*	2.58	0.43	54.85	*g*:*Mortierella*	3.40	3.29	64.77
*o*:*Rhizophlyctidales*	0.20	2.25	66.68	*p*:*Chlorophyta*	1.00	2.55	72.83	*p*:*Chlorophyta*	2.04	3.72	57.44	*p*:*Chlorophyta*	2.87	2.46	66.42
*p*:*Ciliophora*	0.62	2.28	68.28	*p*:*Ascomycota*	1.05	0.34	73.88	*p*:*Ascomycota*	1.53	0.02	59.14	*g*:*Laetisaria*	0.00	1.33	67.89
*f*:*Chitinophagaceae*	4.21	1.74	6.57	*f*:*Chitinophagaceae*	4.41	2.22	5.26	*f*:*Chitinophagaceae*	5.16	3.37	4.96	*f*:*Chitinophagaceae*	3.00	1.42	4.72
*f*:*Comamonadaceae*	2.33	1.40	9.06	*g*:*Niastella*	2.24	0.47	9.66	*g*:*Anaeromyxobacter*	3.37	1.58	9.91	*g*:*Bacillus*	2.76	3.24	7.50
*g*:*Terrimonas*	2.37	3.05	11.02	*f*:*Anaerolineaceae*	3.65	4.88	12.59	*o*:*Burkholderiales*	1.43	0.59	12.25	*g*:*Anaeromyxobacter*	1.39	0.68	9.59
*g*:*Anaeromyxobacter*	1.18	0.58	12.78	*g*:*Bacillus*	2.53	2.50	15.14	*f*:*Clostridiaceae_1*	0.87	0.07	14.48	*f*:*Comamonadaceae*	2.41	1.72	11.64
*g*:*Niastella*	1.22	0.58	14.52	*p*:Thaumarchaeota*	2.68	2.54	17.15	*g*:*Anaeromyxobacter*	0.98	0.33	16.28	*o*:*Burkholderiales*	1.09	0.43	13.59
*f*:*Anaerolineaceae*	2.58	3.20	16.19	*g*:*Streptomyces*	0.91	0.19	18.98	*f*:*Comamonadaceae*	2.68	2.08	17.94	*p*:Thaumarchaeota*	2.90	2.81	15.38
*g*:*Nitrospira*	2.37	2.22	17.55	*f*:*Comamonadaceae*	2.36	1.85	20.34	*g*:*RB41*	1.72	2.32	19.59	*p*:Thaumarchaeota*	2.08	1.96	16.62
*f*:*Chitinophagaceae*	0.99	1.49	18.88	*o*:*Burkholderiales*	1.11	0.56	21.66	*g*:*Flavobacterium*	0.55	0.02	21.04	*g*:*Microvirga*	3.27	2.88	17.84
*f*:*Clostridiaceae_1*	0.52	0.02	20.20	*f*:*Chitinophagaceae*	0.98	0.50	22.93	*g*:*Nitrospira*	1.94	2.41	22.42	*g*:*Angiococcus*	1.76	1.99	18.89
*g*:*RB41*	1.77	2.21	21.48	*g*:*Microvirga*	2.63	2.47	24.19	*g*:*Terrimonas*	2.01	2.51	23.79	*g*:*RB41*	1.00	1.33	19.88

* Phyla from the Archaea kingdom

#### Bacterial community

Generally, the contribution of each OTU was lower in the bacterial community than in the fungal community. This can be attributed to the higher bacterial diversity. Therefore, the total contribution of the ten most relevant bacterial OTUs was <25%. The OTU of the family *Chitinophagaceae* contributed the most to the differences between chitin-amended and non-amended samples under all incubation regimes, being always positively affected by the presence of chitin. The OTU *Niastella* was also positively affected by the presence of chitin under aerobic conditions. The OTU *Anaeromyxobacter* was also positively affected by chitin in anaerobic conditions at both temperature regimes and under aerobic conditions with constant temperature. The OTU Burkholderiales was positively affected by chitin in both aeration regimes under fluctuating temperature. The family *Clostridiaceae_1* was also positively affected by chitin at constant temperature at both aeration regimes. Other OTUs that were dominant in the non-amended samples were Terrimonas (at constant temperature in both aeration regimes incubation regimes) and RB41 at aerobic and constant temperature and at anaerobic and fluctuating temperature.

## Discussion

Under our non-amended field conditions, soil solarization showed potential to suppress FOL in the top 10 cm of the soil as reported in other studies [33, 34]. Chitin as a soil amendment at 0.1%wt did not improve FOL control and it decreased solarization efficacy in the upper soil layer. However, at room temperature, chitin-amended samples did show slight but significant suppression of FOL compared to the non-amended samples. This can be promising for anaerobic soil disinfestation (ASD) where higher temperatures are required to control soil-borne pathogens. Further research with higher concentrations of chitin and longer incubation times are worthy of exploration.

The environmental conditions expected near the soil surface during solarization consist of fluctuating temperatures and low oxygen levels (<5%) [35]. These conditions were simulated in our laboratory experiments and confirmed significantly higher FOL levels in the samples incubated under anaerobic conditions and fluctuating temperature. Optimal radial growth of FOL has been observed at 25°C, being negatively affected at 30°C [6]. This is consistent with lower FOL CFUs values observed in solarized plots and in samples incubated at fluctuating temperature between 30 and 40°C (Fig 2B). The negative effect from this temperature incubation regime was not significant likely due to the short duration of the experiment. The results of our study suggest potential reduction of FOL sensitivity to the temperature due to chitin addition. A change in redox potential may also influenced viability of FOL in the solarized samples. Under aerobic conditions, oxygen is used as a terminal electron acceptor until it is depleted. Thereafter, less favorable terminal electron acceptors are used and redox potential of the soil decreases [36]. Microbial metabolism in a highly reduced environment generates volatile organic compounds [37], organic acids [38], and reduced metals [39], which in aggregate may contribute to a decline in the population of pathogenic fungi. Further experiments are needed to confirm the relationship between soil redox potential and FOL inactivation, specifically in the presence of chitin.

Previous experiments showed chitin, at 20 ton/ha, to be one of the best amendments for control of root-lesion nematode, *Pratylenchus penetrans*, and the soil fungus *Verticillium dahliae*, in comparison to a chemical control of 300 L/ha Metam sodium and untreated control [22]. The reason for the low impact of chitin on FOL may be that the amendment rate, 0.1 wt%, was markedly lower than that of our previous study (0.75 wt%, estimated for a rate of 20 ton/ha at 20 cm for a hypothetical soil bulk density of 1.6 kg/m^3^) [15]. Although the 0.1% amendment rate, as recommended by the manufacturer, did not add to FOL control by solarization, it was enough to show an impact on the soil microbial community. Whereas these changes were not sufficient to suppress FOL populations, other studies have reported that alterations in composition of the soil microbiota are responsible for suppression of pathogens in soil [27]. This discrepancy underscores the need to better understand the relationship between chitin amendment rate and impact on the soil microbial community and plant pathogen suppression.

Our study documented effects of chitin amendments and SBS conditions on FOL population size (CFU/g), but not on effect of disease. Other studies have shown that the abundance of FOL in soil is predictive of the severity of Fusarium wilt, with higher inoculum densities being associated with more severe disease [40]. Therefore, treatments that reduce FOL population size are expected to also reduce the risk of damage from Fusarium wilt. However, severity of disease will also be influenced by susceptibility of the lettuce cultivar being grown [29] and temperature during the growing season [6, 40]. Consequently, there is no definitive relationship between FOL population size and severity of Fusarium wilt.

Chitin amendment significantly decreased fungal diversity in the studied soil when incubated at constant temperature (Fig 3A). In particular, a consistent decrease in abundance of fungi in the phylum Basidiomycota was observed in chitin-amended soil (Table 1). However, the relative abundance of this phylum has been observed to increase after applying reductive soil conditions (equivalent to ASD) to soil amended with maize straw [41]. Chitinolytic microorganisms are common in soil and are capable of decomposing chitin under both aerobic and anaerobic conditions [24]. Soil and the rhizosphere were found to harbor chitinolytic microorganisms, with Actinomycetes being the most abundant [24]. However, this phylum was not within the most abundant phyla (Table 1). Species of *Aspergillus*, *Mucor*, and *Mortierella* are prominent among the chitin-degrading fungi found in soil [24], and *Mortierella* is known to colonize chitinous materials like arthropod exoskeletons [42]. In our study, SIMPER analysis did, indeed, show a sharp increase of an OTU of the genus *Mortierella* in the chitin-amended samples. Although *Mortierella* spp. have been associated with *Fusarium* suppressive soils [43] and particularly to banana Fusarium wilt disease (*F*. *oxysporum f*.*sp*. *cubense*) suppression [44], our study did not correlate *Mortierella* presence with FOL suppression. However, samples incubated in anaerobic and fluctuating temperature conditions showed the lowest relative abundance of *Mortierella* (Table 3) and significantly higher FOL CFUs (Fig 2). This may explain the inability of *Mortierella* to suppress FOL in this study, and highlight the sensitivity of *Mortierella* to the extreme soil conditions occurring during SBS. The presumptively deleterious effects of SBS conditions on the *Mortierella* OTU should be further investigated, as species such as *M*. *elongata* have been shown to play a beneficial role in soil due to their functional capacity to degrade a range of toxic organics, decompose recalcitrant substances, and contribute to pools of long-term stable soil organic matter (C sequestration) [45].

The impact of chitin amendment on the bacterial community was less evident, decreasing the diversity of bacteria in the soil incubated in aerobic and fluctuating temperature conditions (Fig 3B). Huang et al [41] observed that applying ASD to soil amended with maize straw (2% dw) significantly decreased the bacterial community diversity, whereas the fungal community was increased. Other studies have also observed dramatic changes on the soil microbial community structure after ASD with peat and cattle manure (at a rate that increased soil organic matter from 1.2% to 3%)[46] or SBS with anaerobic digestates (1.5% dw) [47] application in soils. In these three studies, the treatments (ASD or SBS) without organic amendments always showed a lower impact on the microbial community supporting the idea of the mild treatment applied in this study due to the low chitin amendment rate (0.1% dw). Soil bacteria with the ability to degrade chitin include species of *Flavobacterium*, *Bacillus*, *Cytophaga and Pseudomonas* [24]. An OTU from the family Chitinophagaceae was observed to be enriched under all incubation conditions. Certain species of the Chitinophagaceae, such as *Chitinophaga pinensis*, can degrade chitin and the family has been described as aerobic or facultatively anaerobic which may explain their presence in SBS amended with chitin [48]. Chitin amendment also showed enrichment of an OTU of the family *Clostridiaceae* in samples incubated at constant temperature. Some *Clostridium* sp. like the strain ChK5, decomposes colloidal chitin and produces acetate and a salt of butyric acid [49]. This is relevant because these acids have been found to have a significant role in suppressing soil pathogens and weeds [15]. The enrichment of these organisms at constant temperature suggests that chitin could have better potential as an amendment at similar temperatures commonly found during ASD, at the optimum amendment rate. Actinobacteria are considered the most prolific source of bioactive antifungal metabolites, including polyketides [43]. Their significant decrease in relative abundance under conditions of constant temperature did not correspond with any significant impact on FOL inoculum densities at constant temperature. Finally, chitin amendment promoted enrichment of *Anaeromyxobacter* which was shown to be beneficial for nutrient uptake in maize seedling; and *Niastella* which is known to promote root growth and in plant defense [50], indicating a potential to improve soil health. Further investigation is warranted into the mechanisms, contributions, and optimal concentrations of chitin as an amendment for use with SBS.

## Supporting information

S1 Table Summary of the statistical parameters of the factorial analysis of incubation treatment and amendment type on the colony forming units of *Fusarium oxyxsporum* f. sp *lactucae* during the field experiment. Non-incubated = ST0; Solarized 0-10cm = SH; Solarized 10-20cm = SL.
(DOCX)

S2 Table Summary of multiway ANOVA of aeration regime, temperature regime and amendment type parameters on log transformed data of colony forming units of *Fusarium oxyxsporum* f. sp *lactucae* under controlled laboratory conditions. Aerobic/Anaerobic; constant temperature (30°C)/fluctuating temperature (30–40°C); non-amended soil/chitin amended soil.
(DOCX)

S3 Table Summary of the statistical parameters of the multiway ANOVA of aeration regime, temperature regime and amendment type on the Shannon diversity index of the fungal and bacterial community in controlled laboratory conditions. Aerobic/Anaerobic aeration regimes; constant temperature (30°C)/fluctuating temperature (30–40°C); non-amended soil/chitin amended soil.
(DOCX)

S4 Table Summary of the P-values of the multiway ANOVA of aeration regime, temperature regime and amendment type on the phylum relative abundance of the fungal and bacterial community in controlled laboratory conditions. Aerobic/Anaerobic aeration regimes; constant temperature (30°C)/fluctuating temperature (30–40°C); non-amended soil/chitin amended soil.
(DOCX)
