# Supplementary material for: Changes of Fusarium oxysporum f.sp. lactucae levels and soil microbial community during soil biosolarization using chitin as soil amendment
Source: PLoS One. 2020 May 5;15(5):e0232662. doi: 10.1371/journal.pone.0232662 (PMC7199936; doi:10.1371/journal.pone.0232662)
Supplement: S1 Table — Non-incubated = ST0; Solarized 0-10cm = SH; Solarized 10-20cm = SL. (DOCX) [file pone.0232662.s001.docx]

Table S1. Summary of the statistical parameters of the factorial analysis of incubation treatment (non-incubated = ST0; Solarized 0-10cm = SH; Solarized 10-20cm = SL) and amendment type on the colony forming units of *Fusarium oxyxsporum* f. sp *lactucae* during the field experiment

| **Term** | **Estimate** | **P-value** |
| --- | --- | --- |
| Model constant | 185.94125 | <.0001* |
| Amendment[Chitin] | -5.73875 | 0.7846 |
| Treatment[SH] | -31.75125 | 0.3852 |
| Treatment[SL] | -113.0313 | 0.0037* |
| Treatment[ST0] | 55.72375 | 0.1321 |
| Amendment[Chitin]*Treatment[SH] | 126.54875 | 0.0014* |
| Amendment[Chitin]*Treatment[SL] | -21.35125 | 0.5580 |
| Amendment[Chitin]*Treatment[ST0] | -27.59625 | 0.4497 |
