# Supplementary material for: Changes of Fusarium oxysporum f.sp. lactucae levels and soil microbial community during soil biosolarization using chitin as soil amendment
Source: PLoS One. 2020 May 5;15(5):e0232662. doi: 10.1371/journal.pone.0232662 (PMC7199936; doi:10.1371/journal.pone.0232662)
Supplement: S2 Table — Aerobic/Anaerobic; constant temperature (30°C)/fluctuating temperature (30–40°C); non-amended soil/chitin amended soil. (DOCX) [file pone.0232662.s002.docx]

Table S2. Summary of Multiway ANOVA of aeration regime, temperature regime and amendment type parameters on log transformed data of colony forming units of *Fusarium oxyxsporum* f. sp *lactucae* under controlled laboratory conditions: Aerobic/Anaerobic; constant temperature (30ºC)/fluctuating temperature (30-40ºC); non-amended soil/chitin amended soil.

| **Term** | **Estimate** | **P-value** |
| --- | --- | --- |
| Model constant | 2.5593522 | <.0001* |
| Amendment[Chitin] | 0.0092612 | 0.6054 |
| Incubation[Aerobic] | 0.0493212 | 0.0336* |
| Incubation[Anaerobic] | -0.015569 | 0.4928 |
| Amendment[Chitin]*Incubation[Aerobic] | -0.015205 | 0.5029 |
| Amendment[Chitin]*Incubation[Anaerobic] | 0.0077296 | 0.7330 |
| Temperature[Constant] | 0.0791716 | <.0001* |
| Amendment[Chitin]*Temperature[Constant30?] | -0.018477 | 0.2434 |
| Incubation[Aerobic]*Temperature[Constant30?] | 0.0283814 | 0.2139 |
| Incubation[Anaerobic]*Temperature[Constant30?] | 0.0507902 | 0.0289* |
