# Supplementary material for: Changes of Fusarium oxysporum f.sp. lactucae levels and soil microbial community during soil biosolarization using chitin as soil amendment
Source: PLoS One. 2020 May 5;15(5):e0232662. doi: 10.1371/journal.pone.0232662 (PMC7199936; doi:10.1371/journal.pone.0232662)
Supplement: S3 Table — Aerobic/Anaerobic aeration regimes; constant temperature (30°C)/fluctuating temperature (30–40°C); non-amended soil/chitin amended soil. (DOCX) [file pone.0232662.s003.docx]

Table S3. Summary of the statistical parameters of the Multiway ANOVA of aeration regime, temperature regime and amendment type on the Shannon diversity index of the Fungal and Bacterial community in controlled lab conditions: Aerobic/Anaerobic aeration regimes; constant temperature (30ºC)/fluctuating temperature (30-40ºC); non-amended soil/chitin amended soil.

| **Term** | **Estimate** | **P-value** |  |
| --- | --- | --- | --- |
| **Fungi** |  |  |  |
| Model constant | 2.8012825 | <.0001* |  |
| Incubation[Aerobic] | -0.122194 | 0.2080 |  |
| Temperature[Const] | 0.1582426 | 0.1083 |  |
| Amendment[Chitin] | -0.263328 | 0.0118* |  |
| Incubation[Aerobic]*Temperature[Const] | 0.065945 | 0.4895 |  |
| Incubation[Aerobic]*Amendment[Chitin] | -0.192445 | 0.0549 |  |
| Temperature[Const]*Amendment[Chitin] | -0.03962 | 0.6766 |  |
| **Bacteria** |  |  | |
| Model constant | 5.9188328 | <.0001* | |
| Incubation[Aerobic] | 0.0068802 | 0.6650 | |
| Temperature[Const] | 0.0117054 | 0.4636 | |
| Amendment[Chitin] | -0.056275 | 0.0022* | |
| Incubation[Aerobic]*Temperature[Const] | 0.0121086 | 0.4486 | |
| Incubation[Aerobic]*Amendment[Chitin] | -0.007667 | 0.6296 | |
| Temperature[Const]*Amendment[Chitin] | 0.0376836 | 0.0273* | |
