# Supplementary material for: Changes of Fusarium oxysporum f.sp. lactucae levels and soil microbial community during soil biosolarization using chitin as soil amendment
Source: PLoS One. 2020 May 5;15(5):e0232662. doi: 10.1371/journal.pone.0232662 (PMC7199936; doi:10.1371/journal.pone.0232662)
Supplement: S4 Table — Aerobic/Anaerobic aeration regimes; constant temperature (30°C)/fluctuating temperature (30–40°C); non-amended soil/chitin amended soil. (DOCX) [file pone.0232662.s004.docx]

Table S4. Summary of the P-values of the multiway ANOVA of aeration regime (anaerobic/aerobic), temperature regime (constant temperature at 30ºC/fluctuating temperature 30-40ºC) and amendment type (non-amended/chitin-amended) on the phylum relative abundance of the Fungal and Bacterial community in controlled lab conditions.

| **Fungi** | **Term** | **Constant** | **Amendment**  **[Chitin]** | **Incubation**  **[Aerobic]** | **Temperature**  **[Const]** | **Amendment[Chitin]**  ***Incubation[Aerobic]** | **Amendment[Chitin]**  ***Temperature[Const]** | **Incubation[Aerobic]**  ***Temperature[Const]** |
| --- | --- | --- | --- | --- | --- | --- | --- | --- |
| Basyciomycetes | Estimate | 37.86115 | -7.906713 | -4.454245 | -4.015423 | -0.993766 | 3.002297 | 3.453645 |
|  | P-Value | <.0001* | 0.0003* | 0.0193* | 0.0324* | 0.5719 | 0.0996 | 0.0613 |
| Chytridiomycota | Estimate | 4.385188 | -0.045652 | 0.131579 | 0.096258 | -0.925914 | -0.548591 | 0.619559 |
|  | P-Value | <.0001* | 0.9302 | 0.8009 | 0.8536 | 0.0892 | 0.3005 | 0.2443 |
| Zygomycota | Estimate | 29.24118 | 13.14404 | 5.213852 | 1.557027 | 5.344303 | -1.488362 | -3.590254 |
|  | P-Value | <.0001* | <.0001* | 0.0440* | 0.5245 | 0.0395* | 0.5427 | 0.1524 |
| Ciliophora | Estimate | 13.12384 | -2.62782 | -0.319936 | 3.61286 | -1.751345 | -0.801513 | -0.076669 |
|  | P-Value | <.0001* | 0.0013* | 0.6463 | <.0001* | 0.0204* | 0.258 | 0.9122 |
| Chlorophyta | Estimate | 8.25718 | -1.049737 | -0.963467 | -1.302171 | -0.582564 | 0.03042 | 0.485898 |
|  | P-Value | <.0001* | 0.0676 | 0.091 | 0.0269* | 0.2937 | 0.9555 | 0.3788 |
| Ascomycota | Estimate | 5.532838 | -1.277831 | 0.540617 | 0.252288 | -0.895814 | -0.106801 | -0.921912 |
|  | P-Value | <.0001* | 0.0499* | 0.3844 | 0.6821 | 0.1573 | 0.8621 | 0.1462 |

| **Bacteria** | **Term** | **Constant** | **Amendment**  **[Chitin]** | **Incubation**  **[Aerobic]** | **Temperature**  **[Const]** | **Amendment[Chitin]**  ***Incubation[Aerobic]** | **Amendment[Chitin]**  ***Temperature[Const]** | **Incubation[Aerobic]**  ***Temperature[Const]** |
| --- | --- | --- | --- | --- | --- | --- | --- | --- |
| Proteobacteria | Estimate | 28.46235 | 1.191399 | -1.041991 | 0.073316 | -0.14134 | 0.544271 | 0.292319 |
|  | P-value | <.0001* | 0.0003* | 0.0009* | 0.7805 | 0.5923 | 0.0508 | 0.2747 |
| Bacteroidetes | Estimate | 16.74429 | 0.458581 | 1.28969 | 1.949956 | 0.256599 | -0.194278 | -1.315028 |
|  | P-value | <.0001* | 0.3092 | 0.0090* | 0.0003* | 0.5653 | 0.6626 | 0.0080* |
| Acidobacteria | Estimate | 10.25458 | -0.898441 | 0.162655 | 1.484257 | -0.106708 | -0.327694 | -0.169112 |
|  | P-value | <.0001* | 0.0004* | 0.4412 | <.0001* | 0.6115 | 0.1305 | 0.4236 |
| Chloroflexi | Estimate | 8.708707 | -0.355115 | -0.034921 | -1.214316 | -0.242374 | 0.111126 | 0.045578 |
|  | P-value | <.0001* | 0.0098* | 0.7782 | <.0001* | 0.0634 | 0.3752 | 0.7134 |
| Actinobacteria | Estimate | 7.199384 | 0.23519 | -0.238185 | -1.13681 | 0.245897 | 0.029823 | 0.476208 |
|  | P-value | <.0001* | 0.1544 | 0.1495 | <.0001* | 0.1376 | 0.8523 | 0.0077* |
| Gemmatimonadetes | Estimate | 6.256318 | -0.374045 | 0.265274 | 0.279661 | -0.136761 | 0.146724 | -0.031865 |
|  | P-value | <.0001* | 0.0020* | 0.0195* | 0.0146* | 0.2014 | 0.172 | 0.7606 |
